# Murine femur micro-computed tomography and biomechanical datasets for an ovariectomy-induced osteoporosis model

**DOI:** 10.1038/s41597-021-01012-8

**Published:** 2021-09-15

**Authors:** Maialen Stephens, Karen López-Linares, Javier Aldazabal, Iratxe Macias, Naiara Ortuzar, Harkaitz Bengoetxea, Susana Bulnes, Natividad Alcorta-Sevillano, Arantza Infante, Jose Vicente Lafuente, Clara I. Rodríguez

**Affiliations:** 1 Vicomtech Foundation, Donostia-San Sebastián, Spain; 2 Grupo de E-Salud del Área de Bioingeniería, Biodonostia, Donostia-San Sebastián, Spain; 3 TECNUN - Universidad de Navarra, Manuel de Lardizábal 13, 20018 Donostia-San Sebastián, Spain; 4 Stem Cells and Cell Therapy Laboratory, Biocruces Bizkaia Health Research Institute, Cruces University Hospital, Plaza de Cruces S/N, 48903 Barakaldo, Bizkaia Spain; 5 University of the Basque Country, UPV/EHU, Leioa, Bizkaia Spain; 6 Laboratory of Clinical and Experimental Neuroscience, Department of Neuroscience, University of the Basque Country, UPV/EHU, Leioa, Bizkaia Spain

**Keywords:** Biomedical engineering, Experimental models of disease

## Abstract

The development of new effective and safer therapies for osteoporosis, in addition to improved diagnostic and prevention strategies, represents a serious need in the scientific community. Micro-CT image-based analyses in association with biomechanical testing have become pivotal tools in identifying osteoporosis in animal models by assessment of bone microarchitecture and resistance, as well as bone strength. Here, we describe a dataset of micro-CT scans and reconstructions of 15 whole femurs and biomechanical tests on contralateral femurs from C57BL/6JOlaHsd ovariectomized (OVX), resembling human post-menopausal osteoporosis, and sham operated (sham) female mice. Data provided for each mouse include: the acquisition images (.tiff), the reconstructed images (.bmp) and an.xls file containing the maximum attenuations for each reconstructed image. Biomechanical data include an.xls file with the recorded load-displacement, a movie with the filmed test and an.xls file collecting all biomechanical results.

## Abstract (summary)

**Table:** 

Measurement(s)	osteoporosis • biomechanical data
Technology Type(s)	micro-computed tomography • biomechanical testing
Factor Type(s)	osteoporosis
Sample Characteristic - Organism	Mus musculus

Machine-accessible metadata file describing the reported data: 10.6084/m9.figshare.15142200

## Background & Summary

Osteoporosis, a skeletal-metabolic disorder, is characterized by decreased bone mass and deteriorated bone microarchitecture, leading to an increased risk of fracture^1,2^. Normally associated with aged populations (postmenopausal women^3^ and elderly men^4^), the high prevalence of osteoporosis is reflected in an estimated 200 million affected people worldwide nowadays^5^. Osteoporosis is a silent disease until the subject experiences a fracture^6^; every year, it is estimated that osteoporosis causes more than 8.9 million fractures worldwide^7,8^. At onset post-menopausal osteoporosis is featured by a loss of trabecular bone, due to the accelerated bone resorption related to a lack of estrogen. Consequently, typical fractures at this time of life involve bones with a high trabecular ratio, e.g. vertebrae and wrist. Later in life, there is also a loss of cortical bone, and as a consequence fractures of the hip, pelvis and long bones (femur, tibia and humerus) are characteristic^9^. Osteoporosis-induced fractures are associated not only with increased morbidity and mortality but also with a high economic burden, being recognized as an increasingly imperative socio-economic concern to be addressed^10^.

Currently, therapeutic treatments for osteoporosis are mainly focused on inhibiting bone resorption as an attempt to increase bone mineral density. However, this delay in the elimination of aged bone compromises bone quality since the old bone is prone to microfractures, which negatively affect its function. Furthermore, the use of these treatments are temporary in order to avoid their long-term adverse effects^11,12^. Thus, improved techniques to assess bone quality are essential for the development of better diagnostic and therapeutic strategies.

Micro-computed tomography (micro-CT), has become an essential tool to evaluate bone quality in terms of microarchitecture by providing high resolution images (of micrometres) which accurately quantify and depict the 3D microarchitecture of both, cortical and trabecular bone^13,14^ parameters proven to correlate well with those obtained from histomorphometry^15^.

In addition to microarchitecture, another crucial parameter to determine bone quality is bone resistance, which is usually measured via the three-point bending mechanical test. This test reports the flexural rigidity of tested bone and the load needed to break it. Due to the combination of biomechanical testing with micro-CT assessment, information of the rigidity of the bone material itself can be elucidated^16,17^. There is only one study determining the rigidity of the bone material in the literature, limiting the rest of them to the assessment of bone rigidity and maximum loads^18^. The fact that one bone is stronger than another, does not necessarily mean that the material is better; greater strength can simply be due to the bone having a larger section or a thicker walls. Knowing the true bone-material resistance is key for comparing different samples despite their varying geometry.

Ovariectomized (OVX) rodent models are used as surrogates of human post-menopausal osteoporosis, resembling the estrogen deficiency context occurring in osteoporotic women. In these models, micro-CT has been a tool of great value to decipher the microarchitecture of osteoporosis^19,20^. Nevertheless, the majority of these micro-CT studies encompass specific trabecular rich regions for analysis (metaphysis of proximal tibia or distal femur), thus, losing information regarding cortical bone.

**Fig. 1:**
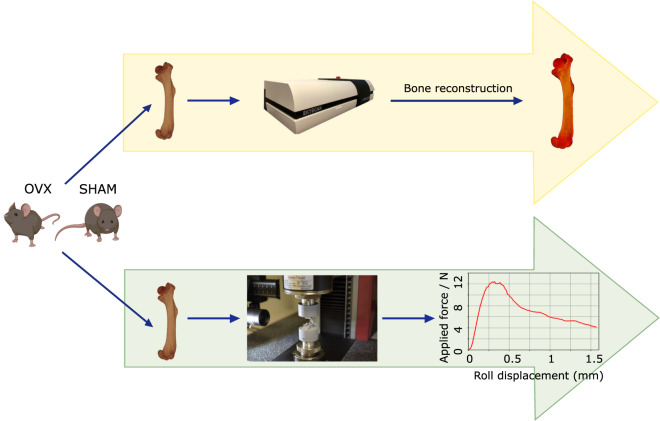
Experimental workflow. Both femurs from C57BL/6JOlaHsd OVX and sham female mice were extracted from each animal; one femur was used for micro-CT measurements and the other for biomechanical testing.

To have a global picture of osteoporotic long bone microarchitecture, we have focused on the micro-CT analysis of the total length of the femur of OVX mice, thus obtaining the maximal information regarding trabecular and cortical bone loss in osteoporotic mice. As controls we have used sham operated female mice (sham), subjected to faked surgical intervention that omits the ovaries removal. Moreover, we have performed biomechanical analysis to the contralateral femur of each sham and OVX female mice, correlating image and biomechanical outcomes. In this way, we have been able to determine bone strength by calculating the real highest mechanical stress that the bone resists (Fig. 1). Hence, to the best of our knowledge, this is the first work that provides a complete dataset of femur micro-CT images for osteoporotic and control (sham) mice correlated with biomechanical information. Given the proven value of this widely used osteoporosis murine model, we have made the micro-CT and biomechanical datasets available in the Figshare open access repository; the data set can be re-used and compared by the scientific community committed to developing new and effective diagnostic, preventive and therapeutic approaches for osteoporosis. In agreement with the culture of sharing scientific knowledge to address health problems and following the principles of 3 Rs (replacement, reduction and refinement) in animal wellness^21^, the data set provided can be very useful in a wide range of purposes. For instance, data can be used to increase statistical power by pooling them with a similar data set, or they can be used to compare the osteoporosis murine model based on C57BL/6JOlaHsd ovariectomized (OVX) mice with other postmenopausal osteoporosis models based on different murine strains. The data could also be useful in generating robust and reproducible methodologies for future femur analysis in mice, and allowing accurate identification of differences between osteoporotic and control subjects, as well as, correlations between the bone geometry obtained from micro-CT with biomechanical tests. To date different approaches in the literature focus on a small region selected ad hoc for density comparisons, while assuming the bone density is homogeneous along different regions of the bone^20,22,23^. Nevertheless, the density variation along the femur may be comparable to the differences between osteoporotic and control mice or between osteoporotic mice that have been treated with different drugs. Hence, our dataset grants an opportunity to define a reproducible protocol for a complete 3D geometry and density analysis that correlates with biomechanical tests, generating a robust way of studying osteoporotic mice femurs and removing the intra- and inter-observer variability derived from the selected region. The 3D images could be re-used to analyse the bone morphology and microanatomy with different quantification algorithms to test algorithms to segment bone and analyse shape/microstructure.

All data shown in this article can be freely downloaded and used by the scientific community. The microCT analyses can be downloaded both as raw data obtained from the microCT equipment and as three-dimensional reconstructions in the form of voxels. These data can be used to analyze geometrical characteristics of healthy or diseased bones. From a biomechanical point of view, the authors make available to the scientific community the complete load-displacement curves obtained in the three-point bending tests, which are not available in the literature. Likewise, we also make available the films of the complete tests, which may be useful for authors who wish to study the failure mode of mouse femurs when bending.

## Methods

### Murine model of osteoporosis

The experimental protocols were reviewed and approved by the Local Ethical Committee for Animal Research of the University of the Basque Country (UPV/EHU, CEEA, ref M20/2019/176). All procedures were performed in accordance with the European Community Council Directive on “The Protection of Animal Uses for Scientific Purposes” (2010/63/EU) and Spanish Law (RD 53/2013) for the care and use of laboratory animals. Briefly, C57BL/6JOlaHsd ovariectomized (OVX) and sham operated (sham) female mice at 8-weeks of age were purchased from ENVIGO. The animals were housed in standard cages in a controlled environment (12-hour light-dark cycles, 21 °C, water and fed powdered rodent diet ad libitum). All animals OVX (n = 7) and sham (n = 8) were euthanized at 17 weeks of age and after isolation and removing of soft tissues, both femurs of each animal were wrapped in saline-soaked gauze; one was frozen at −20 °C and the other at −80 °C until micro-CT and biomechanical analyses were performed, respectively. On the day of analysis, the femurs were thawed at room temperature.

### Micro-computed tomography reconstructions

**Fig. 2:**
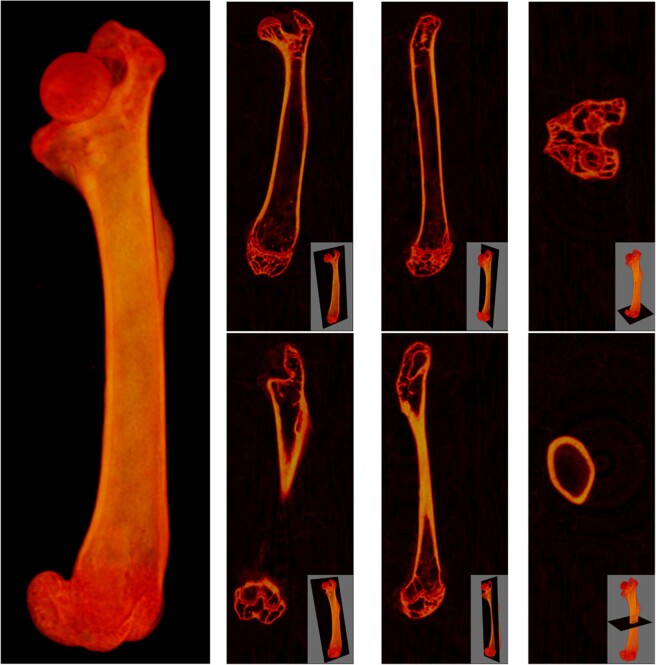
Reconstructed images obtained from the scanning process. Example of the 3D reconstruction of the scan of femur “12 sham”, including sagittal, coronal and axial views at two different positions to show trabecular (top) and cortical (bottom) bone regions.

For the scanning procedure, femurs were placed vertically in a tube sample holder for a stable and symmetrical sample positioning, and they were then fixed in foam to avoid slight movements that may affect scanning at a high resolution. The femur samples were scanned with the SkyScan1172 micro-CT at a voltage of 49 kV and a current of 200 μA using a pixel size of 13.7 μm with a rotation step of 0.3°, a frame averaging of 4, a random movement of 10 and a trajectory of 360°. We employed the aluminum and copper filter and set an exposure time of 2130 ms. For the 3D volume reconstruction, we used the NRecon software developed by Bruker. All images were reconstructed with the optimal reconstruction parameters for bone scans, applying a smoothing of 4, a ring artifact reduction of 10, a beam-hardening correction of 60% and a misalignment compensation adjusted according to the profile window in each case. Regarding the density histogram of the attenuation coefficients, we established the lower limit to be zero and the upper limit was set slightly above the highest value in the brightness spectrum of each bone. The reconstructions were saved as 8-bit Bitmap files, which can be analyzed and processed with the CTAn software by Bruker. Figure 2 shows an example of the final reconstructions.

### Biomechanical tests

**Fig. 3:**
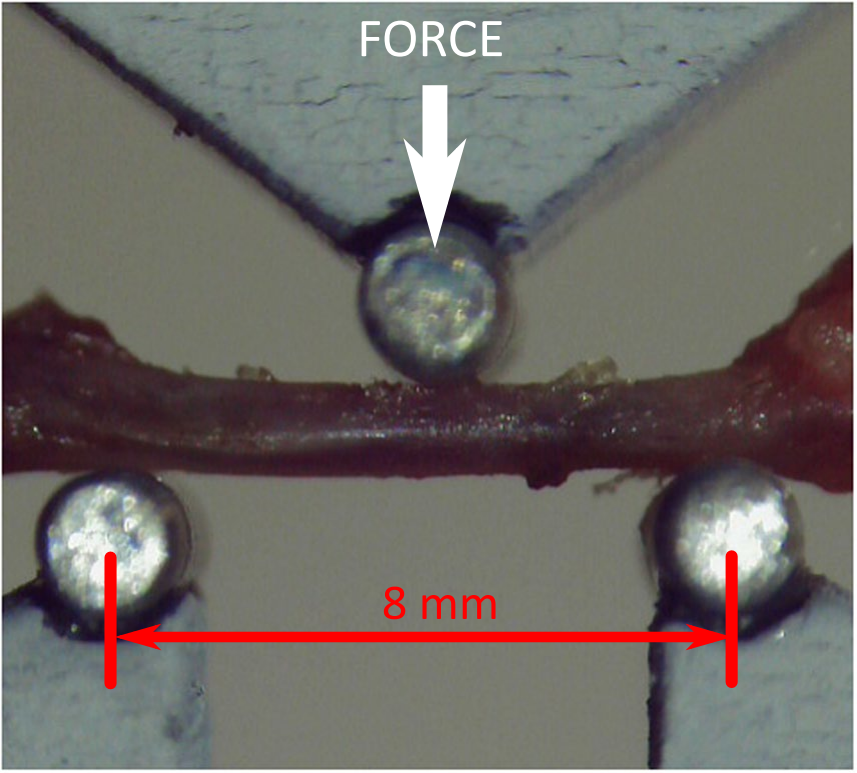
Experimental setup for 3-point bending biomechanical tests. The lower part corresponds to the fixed part of the apparatus. Two 2-millimeter stainless steel rolls are attached to the lower part. Distance between static roll centers is 8 mm. The bone lies on these two rolls. The moving part of the apparatus corresponds to the top roll. The force is measured on this part, as illustrated in the figure. The top 2-millimeter top roll is centered in the setup.

On the day of analysis, the femurs were thawed at room temperature without removing the saline-soaked gauze that retains hydration until the biomechanical tests were performed. Biomechanical properties were obtained using three-point bending flexural test. This test was performed attaching a three-point bend fixture to a universal uniaxial testing machine. The experimental setup consists of a Zwick/Roell machine model ZwickiLine Z1.0 (sn: 734188-2019). The load cell used was a 50 N Zwick/Roell Xforce P (sn: 781140). The software for controlling the machine and recording data was the Zwich/Roell testXpert III v1.4 with all patches installed up to the sx406-218-5. The toll used for performing the three-point bending test was homemade and consists of three stainless steel rolls (diameter 2.0 mm). Two of them are fixed to the lower part of the Zwickiline Z1.0. The axes of these two rods are 8.0 mm apart. The third rod is fixed in the crosshead of the machine and it can move vertically. The moving cylinder is parallel to the others and the horizontal distance between the central cylinder and the two fixed ones is 4 mm, as is shown in Fig. 3.

To compensate tool stiffness, a calibration cycle was performed in testXpert using an extremely stiff Widia bar (Widia THR-F 2.5 × 6.2 × 45.8 mm). This compensation guarantees that displacements recorded during the test correspond to real displacements applied on samples.

For performing biomechanical tests, bones were placed resting on the 2 lower rolls with condyles facing upwards. The positioning of bones was made manually with care to place the bone axis perpendicular to the rolls axis and as centered in the setup as possible. For each experiment, the registered force was zeroed before touching the bone with the upper roll. Once the bone was placed, the upper roll started moving downwards at 1 mm/min until the measured load reached 0.01 N. Once this preload was reached, the system started moving the upper roll downwards at 3 mm/min and recording the measured force and roll displacement.

Tests were filmed using ELP-USBFHD06H 2 megapixels digital camera, controlled by the testXpert software for qualitative inspection. Clips were recorded with a resolution of 480 × 640 pixels at 15 frames per second. The filming started just after reaching the 0.01 N preload.

## Data Records

The generated dataset includes the results obtained from 15 animals divided in two groups based on their conditions: 8 healthy (sham) and 7 osteoporotic (OVX) mice. For each animal a folder was created in the *Figshare* online repository^24^. Each folder contains three files (a first file with the micro-CT acquisition details (.txt), a second file with the load-displacement recorded during the biomechanical test (.xls), and a third file with the filming of the biomechanical test (.avi)) and two folders (one containing the scans directly acquired from the micro-CT in.tiff format and another one with the reconstructed 3D volume as.bmp slices extracted according to the described methodology). Apart from the folder corresponding to each animal, an additional results.xls file is included in the repository, compiling the obtained biomechanical results for all samples and shown in Table 1. The last three columns of the table indicate the microCT slices corresponding to static roll positions and the slice where the load was applied using the top central roll for each sample.

**Table 1:** Biomechanical properties of sham and ovx femurs.

Animal ID.	Max.Load	microCT Slice Number		
	[N]	Left Roll	Load	Right Roll
01ov	12.407	415	707	999
02ov	10.866	334	626	918
03ov	9.788	380	672	964
04ov	14.001	405	697	989
05ov	14.323	428	720	1012
06ov	9.479	406	698	990
07ov	14.172	394	686	978
08sham	14.759	359	651	943
09sham	15.251	419	711	1003
10sham	15.139	414	706	999
11sham	17.220	395	687	979
12sham	14.437	396	687	980
13sham	13.324	411	703	995
14sham	14.902	399	691	983
15sham	14.398	414	706	998

Results from biomechanical tests. The micro-CT slices corresponding to the position where the static rolls were placed and where the loads were applied are indicated in the last three columns.

## Technical Validation

### Micro-CT

Before image acquisition, engineers from Bruker performed maintenance of the micro-CT scanner, ensuring the correctness of the device for subsequent steps. The micro-CT was then aligned with the alignment pin at the scale used to scan all the samples. This pin is used to guarantee that the x-rays project the line around which an object rotates in the 3D space onto the camera as a vertical line. Furthermore, we employed a pair of Bruker-MicroCT BMD calibration rods to carry out density calibration, with concentrations of CaHA of 0.25 and 0.75 g/cm^3^. The calibration led to the following equation for BMD estimation of the reconstructed images:

$$BMD=\frac{AC-0.01549}{0.0616}g/{{\rm{cm}}}^{3}$$

where BMD is the Bone Mineral Density and AC is the attenuation coefficient.

### Biomechanical testing

Before biomechanical determination, the system of the universal axial testing machine and the load cell, was calibrated according to DIN EN ISO 9513:2013-05. The calibration of the system was performed by the laboratory Deusche Akkreditierungsstelle GmbH.

## Usage Notes

Researchers can freely download the study data, which includes micro-CT acquisitions, reconstructions and biomechanical data, from Figshare and use it to perform their own analyses and comparisons.
